# Supplementary material for: Parents’ Knowledge of and Attitude toward Acute Otitis Media and Its Treatment in Children: A Survey at Primary Healthcare Centers in the Aseer Region
Source: Children (Basel). 2023 Sep 19;10(9):1573. doi: 10.3390/children10091573 (PMC10528457; doi:10.3390/children10091573)
Supplement: Supplementary file 1 [file children-10-01573-s001.zip › children-2600249-supplementary.pdf]

## Questionnaire on AOM and its management in children (English)

| **Caregiver survey on the treatment of acute inflammation of the middle ear in children up to the age of 5 years.** | | | |
|---|---|---|---|
| | | | |
| **Do you have a child that is aged up to 5 years?** ☐ Yes ☐ No | | | |
| **We would like to receive some further information about you.** | | | |
| Are you | ☐ Female | ☐ Male | |
| How old are you? | ☐ ☐ ☐ ☐ ☐ | | |
| Single parent | ☐ Yes | ☐ No | |
| You live in | ☐ a more rural environment | ☐ an urban environment. | |
| **Your highest educational degree is** | | | |
| ☐ No educational qualification | | | |
| ☐ Middle school certification | | | |
| ☐ Intermediate high school certification | | | |
| ☐ Final high school certification | | | |
| ☐ University Degree | | | |
| **What kind of health insurance does your child/do your children have?** | | | |
| ☐ Statutory | | | |
| ☐ Private | | | |
| **How old is your child?**<br>☐ Less than 1 year ☐ 1 year ☐ 2 years ☐ 3 years ☐ 4 years ☐ 5 years | | | |
| **Does your child goes to the daycare?** ☐ Yes ☐ No | | | |
| **Does you child uses pacifiers? ?** ☐ Yes ☐ No | | | |
| | | | |

**Below you find statements on the causes and impact of inflammation of the middle ear. How do you appraise these statements? Inflammation of the middle ear…**

| | Fully agree | Mostly agree | Partly agree | Don't really agree | Don't agree at all | Don't know |
|---|---|---|---|---|---|---|
| …is caused by bacteria | | | | | | |
| …is caused by viruses | | | | | | |
| …is associated with intensive earache | | | | | | |
| …is associated with fever | | | | | | |

| | | | | | | |
|---|---|---|---|---|---|---|
| …resolves spontaneously | | | | | | |
| …needs antibiotic treatment | | | | | | |

**Below you find statements on the treatment of pain associated with inflammation of the middle ear. How do you appraise these statements? Best treatment options for earache due to inflammation of the middle ear are…**

| | Fully agree | Mostly agree | Partly agree | Don't really agree | Don't agree at all | Don't know |
|---|---|---|---|---|---|---|
| …medicine with pain relieving / fever reducing substance | | | | | | |
| …antibiotics | | | | | | |
| …naturopathic remedies (e.g. homeopathic globules, herbal medicines) | | | | | | |
| …ear drops with a pain relieving substance | | | | | | |
| …nasal drops with decongestant | | | | | | |
| …household remedies (e.g. onion compresses) | | | | | | |

**Below you find statements on the effectiveness of antibiotics in the treatment of inflammation of the middle ear. How do you appraise these statements? Antibiotics…**

| | Fully agree | Mostly agree | Partly agree | Don't really agree | Don't agree at all | Don't know |
|---|---|---|---|---|---|---|
| …lead to rapid pain relief in children. | | | | | | |
| …lead to rapid fever reduction in children. | | | | | | |
| …generally reduce the likelihood of a relapse of acute inflammation of the middle ear. | | | | | | |
| …generally reduce the risk of permanent ear damage. | | | | | | |
| …negatively affect the children's stomach and bowel. | | | | | | |
| …negatively affect the children's immunity to germs. | | | | | | |
| …may become ineffective after frequent use. | | | | | | |

**With the following statements, we want to find out which contact partners are the most important to you in the case of an inflammation of the middle ear in your child? Please appraise the following statements in regard to your personal attitudes. For the treatment of an inflammation of the middle ear in my child, the opinion of the following person is of major importance to me:**

| | Fully agree | Mostly agree | Partly agree | Don't really agree | Don't agree at all |
|---|---|---|---|---|---|
| General practitioner | | | | | |
| Pediatrician | | | | | |
| Close relatives (e.g. parents, grandparents) | | | | | |
| Parents of other children | | | | | |
| Teachers in child care facilities | | | | | |
| Friends who are health care professionals (e.g. nurses) | | | | | |

**With the following statements, we want to find out which information sources about inflammation of the middle ear in children are the most important to you. Please appraise the following statements in regard to your personal attitudes. Information from the following source is very helpful to me:**

| | Fully agree | Mostly agree | Partly agree | Don't really agree | Don't agree at all |
|---|---|---|---|---|---|
| Newspapers, magazines | | | | | |
| Books | | | | | |
| Radio, television | | | | | |
| Internet | | | | | |

**With the following statements, we want to find out your attitude concerning the "wait and see" treatment strategy with regard to the use of antibiotics in your child with an inflammation of the middle ear. Please appraise the following statements in regard to your personal attitudes.**

| | Fully agree | Mostly agree | Partly agree | Don't really agree | Don't agree at all |
|---|---|---|---|---|---|
| **I am willing to wait** and only use antibiotics when symptoms persist two days. | | | | | |
| **I am willing to wait** and only use antibiotics when symptoms do not improve or even worsen overnight. | | | | | |
| **I am willing to wait.** In the case of persisting symptoms, I consult the medical doctor again before using an antibiotic. | | | | | |
| **I am not willing to wait** and use antibiotics when my child severely suffers from symptoms. | | | | | |
| **I am not willing to wait** and use antibiotics straight away because I am concerned that the disease might get worse. | | | | | |

**Now, we would like to know more about your experiences with inflammation of the middle ear in your child / children. Did you already consult a medical doctor due to an acute inflammation of the middle ear in your child / children?**

| | |
|---|---|
| Yes (then continue with <u>question 8</u>) ☐ | No (then continue with <u>question 12</u>) ☐ |

**If you answered with <u>yes</u>:**

**Please indicate how often your child / children experienced an episode of inflammation of the middle ear?**

- ☐ Less than 3 times
- ☐ 3 to 10 times
- ☐ More than 10 times
- ☐ I don't know

**Which healthcare provider did you consult in the case of an inflammation of the middle ear in your child? Please indicate the healthcare provider you consulted most often.**

- ☐ Pediatrician
- ☐ General practitioner

| | |
|---|---|
| ☐ | Family medicine physcian |
| ☐ | ENT specialist |
| ☐ | First aid pediatrician service |
| ☐ | Emergency service in hospital |

**Below you find a list of drugs that might be used to treat acute inflammation of the middle ear. How often did you ask the medical doctor to prescribe these drugs for your child / children with an acute inflammation of the middle ear?**

| | Always | Often | Sometimes | Rarely | Never |
|---|---|---|---|---|---|
| Medicine with pain relieving / fever reducing substance | | | | | |
| Antibiotics | | | | | |
| Naturopathic remedies (e.g. homeopathic globules, herbal medicines) | | | | | |
| Ear drops with a pain-relieving substance | | | | | |
| Nasal drops with a decongestant | | | | | |

**How often did your doctor prescribe these drugs for your child / children with an acute inflammation of the middle ear?**

| | Always | Often | Sometimes | Rarely | Never |
|---|---|---|---|---|---|
| Medicine with pain relieving / fever reducing substance | | | | | |
| Antibiotics | | | | | |
| Naturopathic remedies (e.g. homeopathic globules, herbal medicines) | | | | | |
| Ear drops with a pain-relieving substance | | | | | |
| Nasal drops with a decongestant | | | | | |

**Many thanks for your support!**
